# Experiences of Using a Digital Guidance and Assessment Tool (the Technology-Optimized Practice Process in Nursing Application) During Clinical Practice in a Nursing Home: Focus Group Study Among Nursing Students

**DOI:** 10.2196/48810

**Published:** 2024-09-10

**Authors:** Hege Mari Johnsen, Andréa Aparecida Gonçalves Nes, Kristine Haddeland

**Affiliations:** 1 Department of Health and Nursing Science, University of Agder Grimstad Norway; 2 Lovisenberg Diaconal University College Oslo Norway; 3 Department of Caring and Ethics, Faculty of Health Sciences, University of Stavanger Stavanger Norway; 4 Department of Health and Nursing Science, University of Agder Kristiansand Norway

**Keywords:** application, assessment of clinical education, AssCE, clinical education assessment tool, electronic reports, feedback, guidance model, smartphone, Technology-Optimized Practice Process in Nursing, TOPP-N, information system success model, nurse, nursing, allied health, education, focus group, focus groups, technology enhanced learning, digital health, content analysis, student, students, nursing home, long-term care, learning management, mobile phone

## Abstract

**Background:**

Nursing students’ learning during clinical practice is largely influenced by the quality of the guidance they receive from their nurse preceptors. Students that have attended placement in nursing home settings have called for more time with nurse preceptors and an opportunity for more help from the nurses for reflection and developing critical thinking skills. To strengthen students’ guidance and assessment and enhance students’ learning in the practice setting, it has also been recommended to improve the collaboration between faculties and nurse preceptors.

**Objective:**

This study explores first-year nursing students’ experiences of using the Technology-Optimized Practice Process in Nursing (TOPP-N) application in 4 nursing homes in Norway. TOPP-N was developed to support guidance and assessment in clinical practice in nursing education.

**Methods:**

Four focus groups were conducted with 19 nursing students from 2 university campuses in Norway. The data collection and directed content analysis were based on DeLone and McLean’s information system success model.

**Results:**

Some participants had difficulties learning to use the TOPP-N tool, particularly those who had not attended the 1-hour digital course. Furthermore, participants remarked that the content of the TOPP-N guidance module could be better adjusted to the current clinical placement, level of education, and individual achievements to be more usable. Despite this, most participants liked the TOPP-N application’s concept. Using the TOPP-N mobile app for guidance and assessment was found to be very flexible. The frequency and ways of using the application varied among the participants. Most participants perceived that the use of TOPP-N facilitated awareness of learning objectives and enabled continuous reflection and feedback from nurse preceptors. However, the findings indicate that the TOPP-N application’s perceived usefulness was highly dependent on the preparedness and use of the app among nurse preceptors (or absence thereof).

**Conclusions:**

This study offers information about critical success factors perceived by nursing students related to the use of the TOPP-N application. To develop similar learning management systems that are usable and efficient, developers should focus on personalizing the content, clarifying procedures for use, and enhancing the training and motivation of users, that is, students, nurse preceptors, and educators.

## Introduction

### Background

During an undergraduate program in nursing, learning in clinical practice, such as a nursing home, is crucial [1,2]. Of the 180 credits required in Norway’s undergraduate nursing program, 90 credits are derived from clinical practice supervised by registered nurses (hereafter referred to as nurses) [3]. Research on nursing students’ learning in clinical practice has shown that access to qualified guidance from nurses is essential to their learning [4-6]. Students have reported dissatisfaction with the limited amount of time spent with nurses in the nursing home setting [7] and have expressed a need for more professional reflection and help from the nurses to develop critical thinking skills [2,8]. Several studies also recommend better collaboration between faculties and nurses to strengthen guidance and assessment to enhance students’ learning in a practice setting [2,5,8-11].

The use of mobile technology is becoming an important part of nursing education [12]. According to Li et al [12], the flexibility and interactivity of mobile apps for learning may positively influence students’ motivations. Further, Shajani et al [13] suggest that digital evaluation tools (in the form of mobile apps or desktop applications) may provide timely and specific in-self-reflection and instructor feedback and are easily accessible. The digital guidance and assessment tool Technology-Optimized Practice Process in Nursing (TOPP-N) was developed to meet some of the challenges described above and is available in app and web versions [14]. Its purpose is to support students, nurses, and faculties in the guidance process in clinical practice, to improve communication and collaboration between the 3 parties [14], to ensure the quality of the students’ achievement of learning outcomes, and to enable following up on them regardless of geographic distance. Importantly, the tool also aims to improve the guidance competence of nurses. Unlike other digital tools to assess students’ achievement in clinical practice [13,15], TOPP-N facilitates the entire guidance and assessment process, not merely the midterm and final assessments.

TOPP-N may be viewed as a learning management system (LMS). From a learner-centered perspective, LMSs are described as platforms that function as mediums that may assist learners to gather, construct, and share knowledge [16,17]. The failure of LMS initiatives in higher-education institutions is often attributed to underestimating the significance of critical factors that influence the success of LMS adoption when designing and evaluating such systems [16,18]. Hence, it is important to identify factors critical to the successful acceptance of an LMS, such as TOPP-N. Perceived usefulness and ease of use are among the main constructs that influence an individual’s behavioral intention and actual use of a system [17,19].

**Figure 1:**
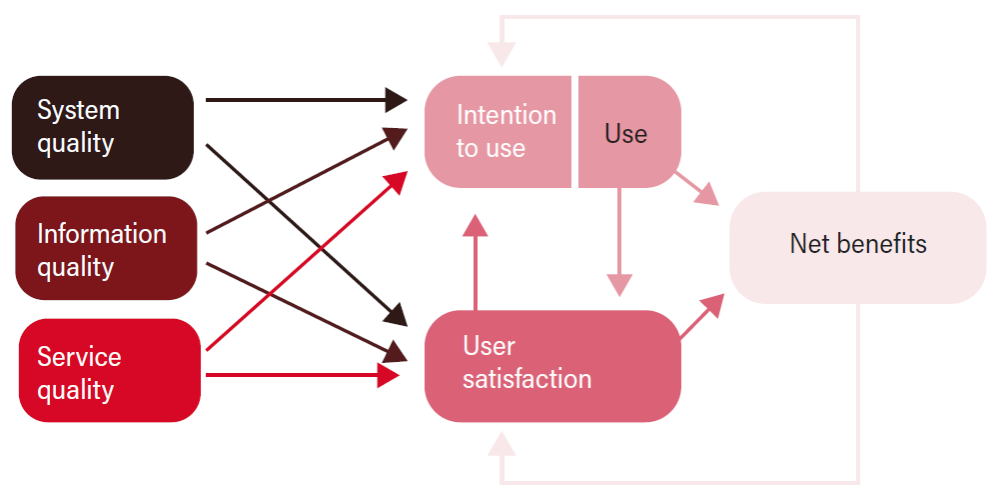
The updated Delone and Mclean Information System Success Model [21].

In our context, we were especially interested in how the students experienced the ease of use, the content, the user support, and the usefulness of the application version of TOPP-N. Thus, the most applicable theoretical framework to our study was the information system (IS) success model developed by Delone and McLean [20] (D&M), which offers a framework of the most critical factors contributing to IS success model in various contexts [17,20]. The framework was first introduced in 1992 but was revised and published in an updated version in 2003 [20]. This framework includes 6 dimensions: system quality, information quality, service quality, use, intention to use, user satisfaction, and net benefits (Figure 1).

System quality indicates desirable characteristics of an IS, such as ease of learning, ease of use (usability), intuitiveness, flexibility, reliability, response times, availability, and desirable characteristics. Information quality indicates the characteristics of system outputs, such as relevance, completeness, accuracy, conciseness, personalization, understandability, timeliness, and security. Service quality refers to the quality of the support that the system users receive from the service providers, which could entail information technology support as well as other types of user support and empathy from staff personnel. Use (behavior) refers to the degree and manner in which the IS or its features are used, for example, the amount, frequency, use patterns, appropriateness, and purpose of use. Intentions to use the system refer to users’ attitudes toward using and reusing the system. User satisfaction refers to users’ overall opinions about the system. Finally, net benefits refer to the benefits resulting from using the IS, such as impacts on individual users, groups, organizations, industries, and nations [20,21].

As shown in Figure 1, the D&M model views IS success as a multidimensional, interdependent construct and uses arrows to describe associations among the dimensions. For example, the quality dimensions (information quality, system quality, and service quality) will singularly or jointly affect subsequent use and user satisfaction. The intention to use and use, user satisfaction, and net benefits dimensions measure IS success or effectiveness. Further, intention to use (attitude) links with use (behavior). According to D&M [20], positive experiences with use will lead to greater user satisfaction and will increase the intention to use. Net benefits emerge because of use and user satisfaction. Whether the experience of benefits is positive or negative, it will lead to increased or decreased intention to use and user satisfaction [20]. D&M [20] recommend that the application context of the D&M model dictate the appropriate specification and application, as well as the various weights assigned to the dimensions of system quality, information quality, and service quality. In this study, *net benefits* describe the individual impacts or perceived usefulness of the app.

In recent reviews of the application of D&M’s IS model [22,23], we have found no other studies that have applied the framework to evaluate a similar app for guidance and assessment of students during clinical practice in health education. Further, there is a lack of qualitative studies based on this framework in an educational context [23]. Hence, the aim of this study was to use the D&M framework to explore first-year nursing students’ experiences of using the TOPP-N application for guidance and assessment in clinical practice in nursing homes.

### TOPP-N Application

**Figure 2:**
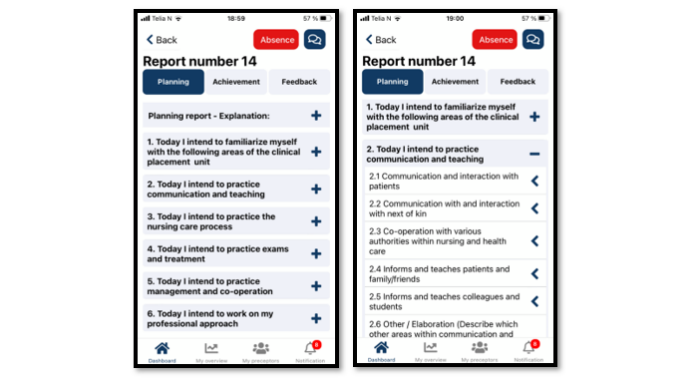
Example of a Technology-Optimized Practice Process in Nursing (TOPP-N) planning report (mobile version screenshot).

**Figure 3:**
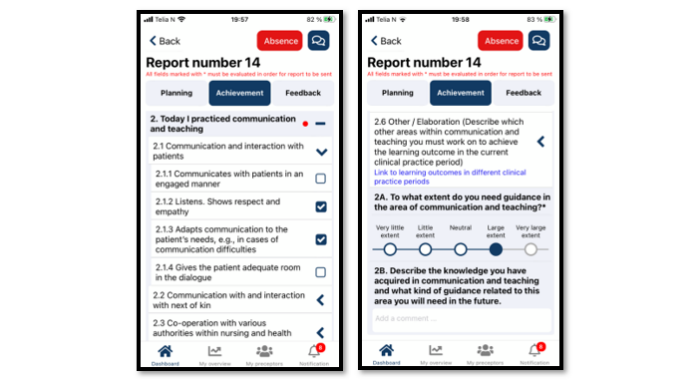
Example of a Technology-Optimized Practice Process in Nursing (TOPP-N) achievement report (mobile version screenshot).

To ensure coherence between learning activities, learning outcomes, and assessment, the processes in TOPP-N are based on constructive alignment [24] and promote metacognition to stimulate nursing students’ development of critical thinking [25]. To achieve this, TOPP-N provides both a guidance module and an assessment module. In the guidance module, the students fill out planning reports (Figure 2) and achievement reports (Figure 3). The former improves students’ awareness of the learning objectives and helps them focus on learning activities that contribute to achieving the learning outcomes. The latter stimulates students to reflect on what they have learned. The content is based on AssCE (Assessment of Clinical Education [26]). AssCE is a research-based and validated assessment instrument.

The reports are stored on a server and, together with the nurses’ daily personal guidance, form the basis for feedback from the nurses for the individual students. A free-text field gives students and nurses the opportunity to elaborate on the answers and communicate directly with one another. The nurse educators at the faculties responsible for the students can access reports and feedback overviews at any time and, consequently, support the students and nurses as necessary. Thus, the guidance during clinical practice is thoroughly documented and provides a better basis for student assessment [27,28]. AssCE [26] is integrated within the assessment module and is used at the students’ midterm and final evaluations. As shown in Textbox 1 [26], AssCE has 21 assessment points under 5 main areas: (1) communication and teaching, (2) the nursing care process, (3) examinations and treatments, (4) work management and cooperation, and (5) professional attitude.

**Figure 4:**
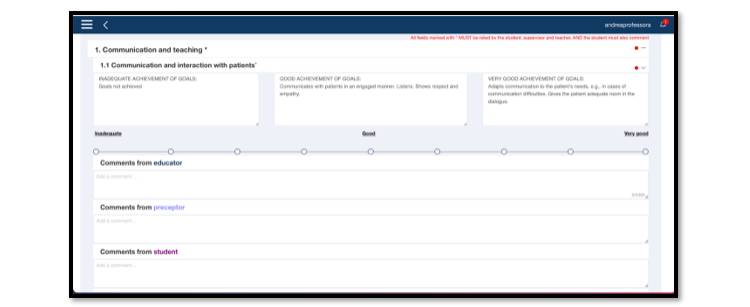
Example of an assessment point in Technology-Optimized Practice Process in Nursing (TOPP-N) with grading and explanatory text (web version screenshot).

Figure 4 shows an example of an assessment point in TOPP-N with grading and explanatory text.

**Main areas and assessment criteria factors in the Assessment of Clinical Education instrument [26].**

**Main areas and assessment criteria**

**I. Communication and teaching**

- Communication and interaction with patients
- Communication with and encounter with family and friends
- Cooperation with various authorities within nursing and health care
- Informs and teaches patients and family or friends
- Informs and teaches colleagues and students

**II. The nursing care process**

- Describes patients’ nursing care needs
- Plans and prioritizes nursing-care interventions
- Carries out nursing-care interventions
- Follows up on needs or problems and nursing-care interventions
- Reports, documents, and record keeping

**III. Examinations and treatments**

- Participates in and carries out examinations and treatments
- Administers medications

**IV. Work management and cooperation**

- Plans, organizes, allocates, and follows up on work assignments
- Cooperates
- Readiness to act
- Safety awareness

**V. Professional approach**

- Scientific awareness
- Ethical awareness
- Self-knowledge
- Thoroughness, reliability, and judgment
- Independence

## Methods

### Study Design and Method

This study used an explorative qualitative design and used focus groups (FGs) to collect data. According to Krueger and Casey [29], FGs can capture the diverse range of participants’ perspectives or feelings about a topic. This method also promotes a synergy that goes beyond individual interviews. The study was conducted in line with the COREQ (Consolidated Criteria for Reporting Qualitative Research) checklist [30] (Multimedia Appendix 1).

### Participants

A convenience sampling method was used to recruit first-year undergraduate nursing students. Before they started using the app in clinical practice, they attended a 1-hour digital course on the app and its use. In addition to receiving course material, the participants had access to instruction videos through the TOPP-N login page and had access to a paper-based brochure with a short user instruction. The participants attended 8 weeks of clinical practice in community services and were placed at 4 different sites (nursing homes or short-term rehabilitation units). During that period, 31 first-year nursing students used the application, and all were invited to participate in the study. The last author contacted the nurse educators (n=4) responsible for the nursing students’ clinical practice, who further invited all the students to participate. A total of 19 students voluntarily participated, and no one who wanted to participate was excluded. The participants had never used the application before the study. Four FGs were conducted, with a different number of participants in each group. One reason for the difference in the number of participants was that the recruitment and interviews were conducted immediately after a reflection group meeting with the students and their nurse educator responsible for their clinical practice. These reflection groups varied in the number of participants. Another reason was that participation in the study was voluntary, and all participants who wanted to participate were included.

### Data Collection

The FGs were conducted in March and April 2022. The first and last authors planned to conduct all the FGs, but the second was conducted by the last author alone for logistical reasons. A semistructured interview guide with 17 primary questions was used (Multimedia Appendix 2 [30]), including demographic questions and questions based on the theoretical framework of D&M. Before the data collection, one coauthor who had contributed to developing the application evaluated the interview guide for relevance and clarity. The guide was also reviewed by nurse educators (other than the ones responsible for the nursing students’ clinical practice) who had contributed to planning the use of the application to follow up on the students’ clinical practice. Because of the reviews, a question about how users compared TOPP-N with other assessment forms was removed, as this was their first clinical placement and they had never used anything but TOPP-N. The interviews were held in meeting rooms on the 2 universities’ campuses. The FGs were audio recorded and lasted from 41 to 62 minutes (52 min on average, SD 10).

### Data Analysis

The last author transcribed all the interviews, which were then subjected to combined inductive and deductive analysis. The first author initially coded all the transcribed data inductively, after which the coded meaning units were sorted according to the categories of the IS success model model by means of directed or deductive content analysis [31,32]. Directed content analysis ideally includes both deductive and inductive analysis [31,32], so the deductive analysis was followed by an inductive process in which subcategories were identified within each predefined category. All the authors read the transcribed data and discussed the findings of the deductive and inductive analyses until they reached consensus. Table 1 provides an example of the analytic process.

**Table 1:** An example from the analysis process.

Meaning unit	Condensed meaning unit	Code	Subcategory	Predefined category
In the reports, you are asked to deal with all the learning outcomes each week. But if it was decided which outcomes to focus on the various weeks, then you didn’t have to deal with everything all the time.	If learning outcomes were predefined for the various weeks, then you would not have to deal with all the outcomes each time.	Desire for predefined areas for the various periods	Amount and relevance of learning objectives	Information quality

### Ethical Considerations

This study was approved by the Norwegian Centre for Research Data (607,252) and followed national ethical research guidelines [33]. All the participants received verbal and written information about the study and FGs in advance and signed informed consent forms before the FGs started. Each participant was assigned a number in case further information was required. To maintain confidentiality, the last author kept the names and participant numbers separately and stored all personal information in a secure location. None of the authors were in a teacher-student relationship with the participants and had not physically met the students before the actual interviews.

## Results

### Findings

The characteristics of the 19 participants in the 4 FGs are shown in Table 2. The participants included 16 women and 3 men, and the median age was 20 years. They were approximately equally distributed between the 2 different campuses, and most of them had attended the digital course about TOPP-N before using it in clinical practice. However, only 6 of the participants had used the instructional videos about TOPP-N. A total of 11 participants used TOPP-N on mobiles and PCs; 7 used it on mobiles only; and 1 participant used TOPP-N on both mobile and PCs and tablets.

**Table 2:** Study participant demographics in the different focus groups (FGs; n=19).

			FG 1 (n=7)		FG 2 (n=3)		FG 3 (n=6)		FG 4 (n=3)
**Sex, n**									
	Female	6		2		5		3	
	Male	1		1		1		0	
Age (y), median (IQR)			22 (19-30)		19 (19-22)		20 (19-24)		20 (20-21)
**University campus**									
	A	0		0		Yes		Yes	
	B	Yes		Yes		0		0	
**Attended digital course, n**									
	Yes	6		2		5		3	
	No	1		1		1		0	
**Used instructional videos, n**									
	Yes	0		1		5		0	
	No	7		2		1		3	
**Use of platform, n**									
	Mobile only	5		0		2		0	
	Mobile and PC	2		2		4		3	
	Mobile, PC, and tablet	0		1		0		0	

Textbox 2 describes the main findings identified in this study. The findings related to each subcategory are further described below. The texts in italics are authentic statements of the respondents.

**Overview of identified subcategories related to each predefined main category of the information systems success model.**

**System quality**

- Ease of use
- Desirable functionality

**Information quality**

- Amount and relevance of learning objectives
- Completeness and understanding of learning objectives

**Service quality**

- Instruction and information
- Follow-up and guidance
- User support

**Use and intention to use the app**

- Patterns of use
- Intention to continue using the app

**User satisfaction**

- Perceived benefits of using the app
- Perceived disadvantages of using the app

**Impact on guidance, assessment, and competence**

- Feedback
- Visualization of competence

### System Quality

#### Ease of Use

Most of the participants who had attended the digital course believed it was easy to learn how to use the app, and some said that they learned to use the app through “learning by doing” and “trial and failure.” As one participant commented, “The application itself is very self-explanatory. If you have tried it once and figured out how to write a report, then you know how to do the next report” (participant 7, FG 1). The few participants who had not attended the digital course (or attended only part of it) found the app difficult to learn and required guidance from fellow students.

The participants perceived that the user interface and use of the app were quite similar on a PC and a mobile phone but mentioned that the text box for writing comments was very small on the mobile app, forcing them to scroll up and down on their mobile when providing comments. In addition, some participants commented that it was cumbersome to switch between the mobile phone and computer, as they could not be logged in at the same time on both platforms. However, the participants described the autosave function as an advantage:

Say that you don’t complete the report...then it is saved until the next time you access it and complete it. I thought that was very good.

Participant 3, FG 3

Regarding completion of the achievement report, some of the students had trouble figuring out how to view the difference in grading achievement between a student and a preceptor. However, most of the participants said that using the app became easier after once using the planning and achievement part of the guidance module and after attending their first midterm evaluation using the assessment module.

#### Desired Functionality

The participants wished that the list of learning objectives would be adjusted depending on their personal achievements. For example, the learning objectives they had ticked off in the planning and achievement report one week would disappear from the planning report’s list of learning objectives the next week:

Now, we start over for each report in the application. And we must base further reports on our own memory in relation to what we have ticked off previously...You can do it systematically yourself, but a tip for further development of the application is that it systematises it for you.

Participant 5, FG 3

With this kind of functionality, they could also observe their progress. As one put it, “It would have been a little more fun if the planning reports were a little shorter each time, because then we might have had a little more motivation to manage to get through everything!” (participant 2, FG 2). The things that they said could disappear from the planning reports included, for example, learning objectives about knowing the physical surroundings and routines at their clinical practice site.

One participant suggested that it should also be possible to receive alerts (ie, reminders to complete the planning and achievement reports on time) without opening the app (as was possible with other apps on their phone).

### Information Quality

#### Amount and Relevance of Learning Objectives

Most of the participants felt that the list of learning objectives in the planning report was useful, as they were reminded of various things they needed to focus on during their clinical practice. However, the list of learning objectives in the planning report was also perceived as overwhelming by many of the participants and, for some, impossible to achieve. Many participants pointed out that not all the learning objectives were relevant to their level and clinical practice.

The participants appreciated the ability to choose an individual number of learning objectives from the list, and many commented that they wished the list of learning objectives in the planning report was predefined according to their level (first year) and included a checklist of the procedures relevant to their clinical practice. It was also suggested that some subitems (eg, under communication) could have been merged into fewer items.

Several participants also mentioned the challenge of planning learning outcomes, as the week never turned out as planned. One participant explained it this way:

Nursing is actually a very unstructured profession. Suddenly, something happens, and you have to change your whole day! Or the plan for your day. So, it was a bit difficult to plan.

Participant 1, FG 4

All the participants found it useful that the learning objectives they had picked for a week were highlighted in yellow in the guidance module’s achievement report, which clarified the objectives requiring comment in the achievement report. The participants also appreciated that they could add new learning objectives to the achievement report, as it often happened that their plan changed or they had performed more activities than planned.

#### Completeness and Understanding of Learning Objectives

Most participants appreciated the ability to add free text, particularly in the planning and achievement reports, as they could specify their learning objectives and explain why other objectives were not relevant. As one participant put it, “The best thing about TOPP-N was that we could write comments” (participant 2, FG 1). Likewise, all the participants appreciated receiving free-text comments from their preceptors in the achievement report, saying that these comments made their preceptor’s evaluation and feedback more specific and complete:

It was good that we received feedback on all the objectives we had ticked off, too. That there was specific feedback, not just summarizing.

Participant 6, FG 3

After filling out the assessment form in the assessment module, many participants experienced that they, their preceptor, and the teacher had somewhat different understandings of some learning objectives, specifically those related to handling acute situations and communicating with patients and their relatives.

In regard to noting the achieved goals in the assessment form, many participants regretted not having the option to choose “not conducted” or “not applicable,” as the only available option was “insufficient.” One participant argued that this term was quite negative and could easily impact students’ self-esteem. It was suggested that users should be able to grade only the achievement based on performed activities instead of providing a grade for all the learning objectives. However, most of the participants said that they appreciated the ability to add a free-text comment in the assessment form to specify their achievement (or lack thereof) during the midterm and final evaluation.

The participants agreed that the number of grading points (9) in the assessment form was appropriate. Many students said that they were motivated by the ability to view the difference in grading of achievement between themselves and the preceptor, as the preceptor generally graded them higher than they graded themselves.

### Service Quality

#### Instruction and Information

Some of the participants said that they had trouble keeping up during the digital course, partly because the lecturer talked a bit fast and they had not yet accessed the application themselves. One participant complained:

“We couldn’t follow along inside the application at the same time. And that made it a little difficult”.

Participant 2, FG 4

Only a few participants had used the available instructional videos (Table 1), the brochure, or the slideshow received through email. One who had read the brochure said, “I read the brochure. Then I understood how to use the reports—that we should plan first, then execute afterwards and then send the achievement report to get feedback” (participant 3, FG 1). Another who had viewed the instructional videos said:

“They were very easy to follow, so I got it [how to use the application] right away”.

Participant 2, FG 2

#### Follow-Up and Guidance

Some of the participants planned their week together with their preceptor and agreed with their preceptor on how many learning outcomes to focus on. As one commented:

We usually sit down with our preceptor at the start of the week and talk a bit about what we want to focus on that week. Doing it this way has worked really well...so she can facilitate what I plan.

Participant 6, FG 3

Not all the participants received feedback on their achievement reports from their preceptor through the application, however. One participant commented:

Our preceptor has not familiarised (herself) with that application at all. She doesn’t know how it works, and, technically, she’s not good. She said she had received training, but still we have not received a response to any of the reports we have written.

Participant 3, FG 2

The participants who received feedback on their achievement reports found weekly written feedback very useful, but one said, “I think it might be a bit too often to have such feedback once a week” (participant 2, FG 3). Some participants noted that their preceptor’s follow-up varied; in 1 week, they might receive comments on some or almost all the learning outcomes in the achievement report, while in another week they received only a summarized comment. The students also observed that the number of comments in the assessment module decreased from the first to the last week, but most participants said that they received comments on all the learning outcomes from their preceptors at their midterm and final evaluations. However, not all the copreceptors had access to the application, which raised obstacles to its use. One said, “I’ve been with a secondary preceptor a lot, but she doesn’t have access to TOPP-N, so she doesn’t really know what I’m up to” (participant 7, FG 1). Furthermore, the participants speculated that inconsistency in follow-up through the application could be explained by the preceptor’s lack of competence in using it, a lack of time to fill in the great number of learning outcomes, or the fact that most communication was in person.

#### User Support

None of the participants required technical support from the application developers, but 2 participants said that their preceptors needed support to access the application:

There were some initial difficulties with getting to log in. It took a while for the preceptor to get started on it.

Participant 5, FG 1

Most participants received support for using the application from fellow students. Three students who lived together during their clinical practice also helped one another understand the general learning outcomes and reminded one another to fill in the achieved learning outcomes.

### Use and Intention to Use the Application

#### Patterns of Use

In regard to the choice of platform, several participants commented that they chose to use a PC the first time they logged in to register and when they had to write a great deal of text in the guidance and assessment module, but all the participants said that they preferred using the application on their mobile phone. As one participant put it:

It was easiest to have it on the mobile. If you’re sitting on the bus on the way home after practice, it’s much easier to pick up your mobile phone than pick up your PC. And, if you have two minutes during your lunch break, you can go in and double-check something, or, if your preceptor asks about something, it’s easy to find out on your mobile.

Participant 7, FG 1

Regarding using the application’s guidance module, most of the participants filled in the 2 related reports (planning and achievement) once a week. Some of the participants filled in the planning reports according to the whole list of available learning objectives, while others chose only the most relevant ones each week. Not all left free-text comments in the planning or achievement reports.

Many participants said that they did not fill in the reports during their days in clinical practice, with one explaining that the priority was spending valuable time on nursing tasks rather than on using the application. Some filled in both reports at the same time, while others stopped using the planning and achievement reports when they noticed that their preceptors did not view them. Other students who did not receive feedback on their achievement reports continued using them for their own sakes, as they provided an overview of goals to focus on.

In most cases, the midterm and final evaluation were done in person in practice, in which case the nurse educator used a computer to view the grading and add a final judgment with comments. Only a few participants used the opportunity to follow the adjustments made by the nurse educator on their own mobile phones, and not everyone knew about this option. One participant who had a digital evaluation appreciated the ability to view the interface and the changes made on the teacher’s computer. All the participants used the application’s assessment module, and they were required to fill out the assessment form to prepare for their midterm and final evaluations, but the amount of free text provided in this context varied among the participants.

None of the participants used the function of sending messages to their preceptor or teacher within the application. They explained that one had to enter the application to use this function, so messages could be easily overlooked if they were not logged in. Therefore, both students and preceptors preferred using text messages on their mobile phones.

#### Intention to Continue Using the Application

Most of the participants indicated that they would continue using the application if the list of learning objectives in the guidance module could be better adjusted to their individual clinical practice and level*.* They also noted that it would be easy to continue using the application when both students and preceptors were more familiar with it.

One participant expressed skepticism about using such an application, arguing that the application should supplement and not replace face-to-face conversations with preceptors. The participant explained that much communication is lost when one relies only on written words.

### User Satisfaction

#### Perceived Benefits of the Application

Several participants remarked that they liked the application’s concept, with one saying, “I really like the concept or idea of TOPP-N” (participant 3, FG 2) and another adding,

I really liked that it was digital! That we didn’t have to “hold a pile of paper” and look after it. It is very easy for papers to disappear or to be destroyed, folded or put somewhere where you can’t find it again. So, it was very good that we had everything on our mobile. I really liked that!

Participant 1, FG 4

Other advantages experienced by the participants included, for example, that the list of learning outcomes in the planning report reminded them of what to focus on in that period. As one participant explained, “I’m a big fan of setting concrete goals, because then it’s much easier to work towards them” (participant 5, FG 3). Because the planned outcomes were visible, both students and preceptors became more conscious of them. In relation to student awareness, one participant commented:

There were actually a lot of items to go through. Both main and subitems. But I feel that, if you spend some time on it, you have better control over what you expect to go through during the week...Then you sort of see what you’ve mastered so far and what you want to improve.

Participant 1, FG 2

The students perceived that, by continually receiving comments or grading before the evaluations, they could better prepare for what was coming and have less fear of possible unpleasant surprises.

#### Perceived Disadvantages of the Application

Among the disadvantages mentioned by the participants was that the list of learning outcomes could be “overwhelming,” “too general,” and “not specific for their practice or level of education.” As one student put it, “What was most difficult about using the application were the learning outcome goals that were there. Because the goals were so big!” (participant 2, FG 4).

Many participants also found it a bit “time-consuming” to fill in all the learning objectives in the planning report, achievement report, and assessment form. The one who had spent the longest time filling out the assessment form said, “I think I spent about one hour or one and a half hours on the report; I got a bit frustrated” (participant 7, FG 1). A disadvantage mentioned by another participant was that relying on an app for guidance and assessment could add screen time and reduce face-to-face communication with the preceptor.

### Impact on Guidance, Assessment, and Competence

#### Feedback

Many students noted that making the planned learning outcomes visible through the application made their preceptor more aware of them. As one participant commented, “Yes...that was an advantage. Because, if we had ticked something off, our preceptor saw it and tried to get us to do it” (participant 3, FG 4). The participants also agreed that using the application provided more comprehensive and continuous feedback and guidance. “In a way, you get a little more follow-up then and a reminder” (participant 4, FG 1). Some pointed out, however, that the application’s usefulness depended on equal commitment from the student and the preceptor. One participant who did not receive feedback on the achievement report said:

“I think I would have gotten even more out of it if our preceptor had been as active as us”.

Participant 3, FG 3

#### Visualization of Competence

Many participants found the application useful for visualizing their own competence: “It is a useful tool in that you have marked which areas you have been through. And it is useful to be able to go through it afterwards” (participant 5, FG 3). The students said that receiving comments or grading from the preceptors on their achievements each week, rather than only on the actual evaluations, made them more aware of their level of competence:

It was reassuring to get feedback during practice and to know a little about how you are doing.

Participant 3, FG 4

Another participant described the personal impact of using the application:

I actually think it was very useful. You sort of got an overview of where both you and your preceptor think you stand. You are often a bit strict with yourself, and then you see that your preceptor means something else. That you can actually do more than you think. So, I felt that, by using the application, you became a little more motivated, and (I) thought that I can actually do this!

Participant 3, FG 3

Similarly, several participants expressed that receiving positive feedback on their competence through the application had a positive impact on their motivation and self-esteem.

## Discussion

**Principal Findings**

This study explored first-year nursing students’ experiences of using TOPP-N for guidance and assessment in clinical practice in nursing homes. The findings are discussed in relation to the categories and their relationships in the D&M model.

### System Quality

Usability, which describes the ease of using a system’s user interface [34], is an important indicator of a system’s quality [20], as a system’s perceived ease of use is among the main elements that influence behavioral intention and actual use of it [17,20]. Most participants in our study found TOPP-N easy to use, but those who had not attended the course struggled a bit when first using it. This was also the result of a feasibility study by Zlamal et al [28], in which some students found it challenging to log into TOPP-N and know what to fill out and when. Based on the findings of this study, we recognize that the available sources of information in the application should be made more visible in an updated version. This aligns with Nielsen’s [34] suggestion for user interface design: “Information required to use the design should be visible or easily retrievable when needed.” A system’s ability to provide desired functionalities and characteristics is another important element of usability [20,34]. The participants noted some functionalities that the application lacked, including the recall and removal of learning objectives that they had already reached, the ability to place an icon on their phone screen (as with other phone apps) to alert them about messages from the nurse preceptor or nurse educator, and the ability to be simultaneously logged in on multiple platforms. According to Nielsen [34], allowing personalization and tailoring of content makes user interfaces more flexible and efficient to use. In light of developments in artificial intelligence and machine learning, it should be possible to personalize the list of learning objectives, but the ability to log in at the same time on different platforms is limited by data security requirements. However, all the students appreciated the flexibility of having TOPP-N on their phone, as it could be used wherever and whenever they wished without their computer. Hence, despite the lack of some desired functionalities, our findings support a positive relationship between system quality and user satisfaction, in line with D&M [20] and Petter et al [21].

### Information Quality

As in the study of Zlamal et al [28], we identified some misunderstandings related to using the planning function of TOPP-N. For example, some participants believed that they had to fill in the whole list of available learning objectives offered by the application when planning or evaluating their achievement in TOPP-N. However, participants appreciated the ability to choose an individual number of learning objectives from the list and write individual comments. This ability to personalize the list of learning objectives is in line with Nielsen’s [34] user interface design principles. One advantage of TOPP-N is that students can individualize the list of learning objectives and implement tailored learning activities and assessment criteria, supporting both constructive alignment—to ensure coherence between learning activities, learning outcomes, and assessment [24]—and TOPP-N’s metacognition approach, which aims to stimulate nursing students’ development of critical thinking [25]. Critical thinking is a crucial skill in a constantly changing profession that deals with the complexity of modern health care demands [27].

Many students wished that the list of learning objectives in the planning report had been predefined according to their clinical practice context and level of education (first year). Their suggestions are in line with information quality indicators, such as “relevance” and “timeliness,” as described by D&M [20] and Petter et al [21]. Considering the rapidly growing older population’s increased need for complex medical and palliative care [6], learning objectives better adjusted to the nursing home context would address the growing need for nursing competence in caring for older adults. However, one advantage of having common learning objectives in TOPP-N is that the application is applicable in many different contexts. In addition, providing common learning objectives promotes the achievement of the desired learning outcomes and the expected competence level for each clinical practice. Furthermore, adjusting the list of learning objectives to a specific educational level would ignore the fact that nursing students’ individual levels of knowledge, skills, and experience may differ.

The participants also wished that the application included a checklist of the procedures relevant to their clinical practice, but Engström et al [35] argue that, if an assessment tool is used more as a checklist, it will have little value for enhancing students’ learning. In other words, if clinical skills or tasks dominate, there is a risk of losing focus on caring-related knowledge and understanding. However, the various clinical placements can provide students with checklists as a supplement to the content in TOPP-N.

According to D&M [20] and Petter et al [21], “completeness” and “accuracy” are 2 important indicators of a system’s information quality. As in the study of Aase et al [10], some of our participants experienced that students, preceptors, and teachers had different understandings of some learning objectives and concepts. According to Nielsen [34], an IS’s words, phrases, and concepts should be familiar to users, but the challenge of changing the phrases and content in TOPP-N is that the application is based on the assessment tool AssCE, whose purpose is to promote a standardized language and content [35]. A potential solution could be for nurse educators, nurse preceptors, and nursing students to agree on the included concepts and phrases before using the application as demonstrated by the participants in Aase et al [10]. Otherwise, the participants seemed satisfied with the information quality in TOPP-N.

### Service Quality

Service quality embraces all the support that system users receive when using the application [20,21]. Our findings indicate that the digital course about the TOPP-N application should include a practical part where the students can follow along inside the app during the course. In addition, the link to available instruction videos should be made more visible.

Qualified guidance from nurses is essential to nurse students’ learning [4-6], and good collaboration between students, preceptors, and nurse educators is essential to achieving a sound, objective assessment of students’ learning outcomes [10,35]. As in the study of Zlamal et al [28], our participants experienced that some preceptors had little knowledge and competence in using TOPP-N. The students perceived this negatively and suggested that training for those who use TOPP-N should be improved, especially in cases involving more than one supervisor. The need for additional time for training in the use of digital evaluation tools among nursing instructors was also pointed out in the study of Shajani et al [13]. Furthermore, our findings show that the guidance and support (or lack thereof) received from their nurse preceptors influenced students’ motivation, self-effort, and perceived usefulness. This is in line with Frøiland et al [6], who propose that the supervisory relationship and the role of the nurse preceptor are of utmost significance in influencing students’ learning experiences. Thus, in contrast to previous studies of the D&M model [22], ours shows that service quality was particularly important to the student’s use of the application, user satisfaction, and perceived usefulness. As suggested by Jeyaraj [22], this may be explained by the context in which the model was applied (nursing education) and the specific service provision (guidance and support). Our findings support evidence [6] that suggests that targeted efforts to enhance mentorship practices in nursing homes are needed to maximize the learning potential in this context.

### Use and Intention to Use

The participants in our study preferred to use TOPP-N on their mobile phone, as they could log in at all times, and access was perceived as faster. This accords with the research of Zlamal et al [28], Li et al [12], and Shajani et al [13], who found that using mobile apps is easy to access and can increase students’ motivation for self-study. However, our participants’ choice of platform depended on the task to be done. For example, a computer was preferred during the midterm and final assessments to obtain a better overview of the student’s achievement.

The use of the planning reports varied among the participants and was influenced by the feedback on the reports (or lack of feedback). As in the Zlamal et al [28] study, our participants’ own motivation or motivation by others (nurse preceptors, nurse educators, or fellow students) was an important facilitator of their intention to use the application. This aligns with the theory of adult learning [36], which proposes that learning is dependent on both intrinsic and extrinsic motivation. It also supports the theory of user acceptance of technology [17], which proposes that user acceptance of new technology can be influenced by personal factors (eg, self-efficacy, experience, and computer anxiety) as well as environmental ones (including social influence). Hence, the findings indicate that it is important that nurse educators and faculty facilitate increased motivation and proper use of the application among the nurse students and preceptors. As noted by D&M [20], a system’s use must be appropriate to achieve the expected impacts. Proper use could be enhanced if the guidelines for use better match what is manageable in a busy, everyday routine in clinical practice.

### User Satisfaction

Overall, the participants were quite satisfied with the app, but, like those in the Zlamal et al [28] study, our students expressed that using TOPP-N was quite time-consuming, which was experienced as a disadvantage. According to Nielsen [34], the time required to engage with a digital program (ie, efficiency) is a usability attribute that influences motivation and the intention to use the program. TOPP-N’s efficiency can probably be improved by better personalizing the application’s content, as suggested by the participants. Personalization of the list could also make it easier for the nurse preceptors to obtain an overview of students’ learning objectives, which could improve the usability of the application and increase the preceptors’ intention to use it.

In line with findings from Jeyaraj’s [22] critical metareview, all 3 D&M quality dimensions (system quality, information quality, and service quality) seemed to impact our participants’ satisfaction with the application. Confirming the finding of Li et al [12], however, the students expressed that the application should be a supplement and not a replacement, and TOPP-N aims to be a supplement that enhances the quality of guidance and assessment during clinical practice [14].

### Perceived Usefulness

Learning at clinical practice sites, such as a nursing home, is vital [1,2], and self-awareness, or thinking about one’s own thinking (metacognition), is an important element in bridging knowledge and cognition in learning [37]. Because TOPP-N is based on the concepts of metacognition and critical thinking [14], we were happy that the students recognized that, by making the planned learning outcomes visible, the application made both students and their supervisors more conscious of them. By doing so, it enabled continuous, comprehensive feedback and guidance from preceptors. These findings are in line with research [15,27,28,35] showing that using AssCE provides superior continuity in student guidance and assessment and supports students’ clinical learning. Our findings also indicate that the information quality of a system, such as TOPP-N, is strongly associated with individual impacts [20,21]. In addition, our findings support the notion that ongoing feedback during clinical practice (formative assessment) is fundamental to students’ professional development, as it provides direction and nurtures confidence, motivation, and self-esteem [10,14].

Perceived usefulness is among the main elements that influence individuals’ behavioral intentions and actual use of a system [17,19,20]. Our findings show that the perceived usefulness of the application depended on equal commitment from the student and the preceptor, which aligns with the D&M model [20,21] and research [5,10,11,35] and suggests that service quality—regarding collaboration with nurse preceptors (or its absence)—crucially influences individual impacts, such as students’ clinical learning outcomes. Our findings also imply that nurse students’ experiences of TOPP-N’s positive or negative outcomes will increase or decrease their intention to use it, as well as their user satisfaction [20]. Thus, our findings support the D&M success model [20,21], suggesting that the 6 dimensions of success are interrelated rather than independent, with each dimension required to ensure the perceived usefulness or success of TOPP-N. However, nursing students’ learning in clinical practice may also be influenced by the characteristics of the nursing home’s ward environment, such as the pedagogical atmosphere, management support, and qualified nurse preceptors [6]. This was not investigated in this study.

### Strengths and Limitations of This Study

The values of trustworthiness in qualitative analyses, such as credibility, dependability, and transferability [38], were protected by our chosen qualitative design. To ensure credibility and dependability, a validated IS success model was used to explore nursing students’ experiences of using a mobile app (TOPP-N) for guidance and assessment during clinical practice in a nursing home. To capture the diversity of participants’ feelings and perspectives on the application, FGs were chosen for data collection. However, FGs may have limitations [29]. For example, some participants may comment on all questions, while others may comment several times on a single issue. The FGs had a different number of participants, and the sample size in each interview may have influenced how much each participant contributed to the data collection. We took this into consideration during the analysis and when presenting the findings by ensuring a balanced presentation of experiences and comments from the participants. The total sample of 19 participants provided substantial insights into the perceptions of the participants, and we reached a point where we had sufficient data to draw the necessary conclusions [39].

The main categories of the directed analysis were based on the dimensions of the D&M model. Aware that using a directed approach can blind researchers to the possible presence of other main categories in the text [31], the authors kept an open mind during the analysis process so as not to overlook other main categories or subcategories in the data. All the authors were involved in the discussion and in reaching consensus, but no additional main categories were identified.

Regarding trustworthiness and transferability, the authors have described the context, participants, and research process thoroughly, making it possible to replicate the methodology in similar studies. All the authors are female academics in nursing education and have expertise in qualitative analysis.

A limitation of the study that influenced participants perceptions of the usefulness of TOPP-N was that some preceptors had not familiarized themselves with the application. In addition, not all the copreceptors managed to access the application, which raised obstacles. All preceptors were invited to attend a 1-hour digital course about TOPP-N and its use. In addition, they received the video recording of the course and information through email. The brochure was also available at the 4 clinical practice sites. In retrospect, we recognize that we should have asked for confirmation that the preceptors had viewed the course material and the available information.

### Conclusions

The aim of using TOPP-N as a guidance and assessment tool is to increase the quality, flexibility, and efficiency of clinical practice in the undergraduate program in nursing [14]. Based on the findings, it appears that the application has the potential to improve users’ quality of learning by enhancing metacognition and critical thinking skills. However, the perceived usefulness depends on students’ own motivation and the collaboration and support of nurse preceptors. Students found using the mobile app for guidance and assessment to be very flexible, but they were less satisfied with the amount of time it took to fill in the reports. Improvements should focus on personalizing the content, clarifying procedures for use, and enhancing the training and motivation of both students and preceptors to make the application more efficient.

The application and its implementation process will be improved based on the findings of this study before it is used in other contexts and in further research (ie, a qualitative study with a focus on mental health). Our findings can alert developers and researchers in other educational contexts to the critical factors in the successful acceptance of similar LMSs.

## Appendix

Multimedia Appendix 1 The COREQ (Consolidated Criteria for Reporting Qualitative Research) checklist.

Multimedia Appendix 2 Interview guide.

## Abbreviations

AssCE: Assessment of Clinical Education
COREQ: Consolidated Criteria for Reporting Qualitative Research
D&M: DeLone and McLean
FG: focus group
IS: information system
LMS: learning management system
TOPP-N: Technology-Optimized Practice Process in Nursing

## Data Availability

The qualitative data set is in Norwegian and is not publicly available to protect the anonymity of the participants. On a reasonable request, data may be available from the corresponding author. In that case, the integrity and anonymity of the participants will be maintained.
