# Supplementary material for: Experiences of Using a Digital Guidance and Assessment Tool (the Technology-Optimized Practice Process in Nursing Application) During Clinical Practice in a Nursing Home: Focus Group Study Among Nursing Students
Source: JMIR Nurs. 2024 Sep 10;7:e48810. doi: 10.2196/48810 (PMC11422751; doi:10.2196/48810)
Supplement: Multimedia Appendix 1 [file nursing_v7i1e48810_app1.pdf]

Multimedia Appendix 1

Manuscript: Nursing Students’ Experiences of Using the digital Guidance and Assessment Tool TOPP-N during Clinical Practice in a Nursing Home: A Qualitative Study

Consolidated criteria for reporting qualitative studies (COREQ): 32-item Checklist

Developed from:

Tong A, Sainsbury P, Craig J. Consolidated criteria for reporting qualitative research (COREQ): a 32-item checklist for interviews and focus groups. *International Journal for Quality in Health Care*. 2007. Volume 19, Number 6: pp. 349 – 357

**No. Item**	**Guide questions/Description**	**Reported on Page #**
**Domain 1: Research team and reflexivity**		
Personal Characteristics		
1. Interviewer/facilitator	Which author/s conducted the interview or focus group?	Page 8
2. Credentials	What were the researcher’s credentials? e.g. PhD, MD	On the title page
3. Occupation	What was their occupation at the time of the study?	Page 25
4. Gender	Was the researcher male or female?	Page 25
5. Experience and training	What experience or training did the researcher have?	Page 25
Relationship with participants		
6. Relationship established	Was a relationship established prior to study commencement?	Page 9
7. Participant knowledge of the interviewer	What did the participants know about the researcher? e.g. personal goals, reasons for doing the research	All participants received written information about the study before they gave their consent
8. Interviewer characteristics	What characteristics were reported about the interviewer/facilitator? e.g. Bias, assumptions, reasons and interests in the research topic	Page 25

**Domain 2: study design**		
Theoretical framework		
9. Methodological orientation and Theory	What methodological orientation was stated to underpin the study? e.g. grounded theory, discourse analysis, ethnography, phenomenology, content analysis	Page 7
Participant selection		
10. Sampling	How were participants selected? e.g. purposive, convenience, consecutive, snowball	Page 7-8
11. Method of approach	How were participants approached? e.g. face-to-face, telephone, mail, email	Page 7-8
12. Sample size	How many participants were in the study?	Page 7 and 9 and Table 3 (page 9)
13. Non-participation	How many people refused to participate or dropped out? Reasons?	Page 7-8
Setting		
14. Setting of data collection	Where was the data collected? e.g. home, clinic, workplace	Page 8
15. Presence of non-participants	Was anyone else present besides the participants and researchers?	Page 8
16. Description of sample	What are the important characteristics of the sample? e.g. demographic data, date	Page 7 and 9 and Table 3 (page 9)
Data collection		
17. Interview guide	Were questions, prompts, guides provided by the authors? Was it pilot tested?	Page 8 and Appendix 2
18. Repeat interviews	Were repeat interviews carried out? If yes, how many?	No
19. Audio/visual recording	Did the research use audio or visual recording to collect the data?	Page 8

20. Field notes	Were field notes made during and/or after the interview or focus group?	No
21. Duration	What was the duration of the interviews or focus group?	Page 8
22. Data saturation	Was data saturation discussed?	Page 24-25
23. Transcripts returned	Were transcripts returned to participants for comment and/or correction?	No
**Domain 3: analysis and findings**		
Data analysis		
24. Number of data coders	How many data coders coded the data?	Page 8 and 25
25. Description of the coding tree	Did authors provide a description of the coding tree?	Page 8 and Table 2 page 9
26. Derivation of themes	Were themes identified in advance or derived from the data?	Page 8, 25 and Table 2 (page 9) and Table 4 (page 11)
27. Software	What software, if applicable, was used to manage the data?	Not applicable
28. Participant checking	Did participants provide feedback on the findings?	No
Reporting		
29. Quotations presented	Were participant quotations presented to illustrate the themes/findings? Was each quotation identified? e.g. participant number	Page 11-19
30. Data and findings consistent	Was there consistency between the data presented and the findings?	Page 11-19, and Table 2 (page 9) and Table 4 (page 11)
31. Clarity of major themes	Were major themes clearly presented in the findings?	Page 11-19, and Table 4 (page 11)
32. Clarity of minor themes	Is there a description of diverse cases or discussion of minor themes?	Page 11-19, and Table 2 (page 9) and Table 4 (page 11)
