# Supplementary material for: Experiences of Using a Digital Guidance and Assessment Tool (the Technology-Optimized Practice Process in Nursing Application) During Clinical Practice in a Nursing Home: Focus Group Study Among Nursing Students
Source: JMIR Nurs. 2024 Sep 10;7:e48810. doi: 10.2196/48810 (PMC11422751; doi:10.2196/48810)
Supplement: Multimedia Appendix 2 [file nursing_v7i1e48810_app2.pdf]

Multimedia Appendix 2

Interview guide

Demographic questions:

1. Age?
2. Previous experience with using TOPP-N?
3. Which campus do you belong to (X or Y)?
4. What kind of training did you receive before using TOPP-N?
5. Did you use the instructional videos? If not, why? If so, were they helpful?

Use of TOPP-N:

6. On what platform(s) did you use TOPP-N (on mobile, tablet, PC)? (Any preferences?)
7. How did you experiences using TOPP-N (The technology; system quality/ease of learning/user friendliness etc.)?
8. How did you experience using the planning reports in TOPP-N? (usability and content)
9. How did you experience using the achievement reports in TOPP-N? (usability and content)
10. How did you experience receiving feedback from the nurse preceptor on the reports in TOPP-N?
11. Did you send messages to the nurse preceptor or nurse educator through TOPP-N? If so, how did this work?
12. How did you experience using the assessment form in TOPP-N? (usability and content)
13. How did you experience using TOPP-N in conduction of the mid- and final evaluation?
14. Did you receive any support in use of the application?
15. How is your general satisfaction with TOPP-N?
16. Will you continue using the application?
17. Has use of TOPP-N for guidance and assessment in clinical placement been useful in any way? How?
